# Medium-Dependent Antibacterial Properties and Bacterial Filtration Ability of Reduced Graphene Oxide

**DOI:** 10.3390/nano9101454

**Published:** 2019-10-13

**Authors:** Alexander Gusev, Olga Zakharova, Dmitry S. Muratov, Nataliia S. Vorobeva, Mamun Sarker, Iaroslav Rybkin, Daniil Bratashov, Evgeny Kolesnikov, Aleš Lapanje, Denis V. Kuznetsov, Alexander Sinitskii

**Affiliations:** 1 Research Institute for Environmental Science and Biotechnology, Derzhavin Tambov State University, Tambov 392000, Russia; nanosecurity@mail.ru (A.G.); olgazakharova1@mail.ru (O.Z.); 2 National University of Science and Technology “MISIS”, Moscow 119991, Russia; dmitrymrtv@gmail.com (D.S.M.); kolesnikov@misis.ru (E.K.); dk@misis.ru (D.V.K.); 3 Department of Chemistry, University of Nebraska—Lincoln, Lincoln, NE 68588, USA; nataliia.vorobeva@huskers.unl.edu (N.S.V.); msarker39@huskers.unl.edu (M.S.); 4 Remote Controlled Theranostic Systems Lab, Saratov State University, Saratov 410012, Russia; rybkin.yaroslav@yandex.ru (I.R.); dn2010@gmail.com (D.B.); lapanje.ales@gmail.com (A.L.); 5 Jožef Stefan Institute, 1000 Ljubljana, Slovenia; 6 Nebraska Center for Materials and Nanoscience, University of Nebraska—Lincoln, Lincoln, NE 68588, USA

**Keywords:** graphene oxide, nanotoxicity, antibacterial properties, *Escherichia coli*

## Abstract

Toxicity of reduced graphene oxide (rGO) has been a topic of multiple studies and was shown to depend on a variety of characteristics of rGO and biological objects of interest. In this paper, we demonstrate that when studying the same dispersions of rGO and fluorescent *Escherichia coli* (*E. coli*) bacteria, the outcome of nanotoxicity experiments also depends on the type of culture medium. We show that rGO inhibits the growth of bacteria in a nutrition medium but shows little effect on the behavior of *E. coli* in a physiological saline solution. The observed effects of rGO on *E. coli* in different media could be at least partially rationalized through the adsorption of bacteria and nutrients on the dispersed rGO sheets, which is likely mediated via hydrogen bonding. We also found that the interaction between rGO and *E. coli* is medium-dependent, and in physiological saline solutions they form stable flocculate structures that were not observed in nutrition media. Furthermore, the aggregation of rGO and *E. coli* in saline media was observed regardless of whether the bacteria were alive or dead. Filtration of the aggregate suspensions led to nearly complete removal of bacteria from filtered liquids, which highlights the potential of rGO for the filtration and separation of biological contaminants, regardless of whether they include live or dead microorganisms.

## 1. Introduction

Graphene and related materials are widely recognized for their potential for a multitude of biomedical applications, which include biosensing, bioimaging, anti-cancer therapy, cell growth, tissue engineering and antibacterial agents among others [1,2,3]. Among other materials from the graphene family, graphene oxide (GO) and its reduced form, reduced graphene oxide (rGO), are of particular interest for biomedical applications because of their high surface area, solubility in a variety of solvents—including water and aqueous solutions—and multiple opportunities for surface functionalization [4]. However, while GO, rGO and other graphene-based materials have been widely used in biomedical research [1,2,3,4,5], there are different views on their nanotoxicity [5]. There was a considerable amount of in vitro and in vivo research on toxic effects associated with graphene-based materials [4,5]. Several studies showed that graphene and its derivatives are toxic for microorganisms [6,7,8] and some of the proposed mechanisms of toxicity involve membrane damage and oxidative stress [9,10]. Other authors argue that graphene-based materials do not exhibit inhibitory effects on bacteria [11,12,13,14].

The breadth of results obtained in various nanotoxicity experiments involving graphene and related materials suggests that generalized conclusions should be avoided as safety risks associated with graphene-based materials depend on a large number of factors [4,5]. These factors include the specific type of graphene-based material (GO, rGO, few-layer graphene, graphene quantum dots, etc.), the specific type of biological objects used in a particular study (cells, bacteria, various multicellular microorganisms) and the experimental conditions. The toxicity of graphene-based material of a certain kind, such as GO or rGO, can further depend on the flake size, surface functionalization and concentration, as was demonstrated in multiple studies [15,16,17,18]. 

In this work, we discuss another variable that should be added to the already multidimensional space of parameters that need to be considered in nanotoxicology experiments involving graphene-based materials. We show that in the experiments involving the same dispersions of rGO and same recombinant *Escherichia coli* (*E. coli*) bacteria expressing green fluorescent protein (GFP) the outcome of the nanotoxicity experiment strongly depends on the culture medium. The addition of rGO inhibited the growth of bacteria in a nutrition broth (NB) medium but showed little effect on the behavior of *E. coli* in a physiological solution (PS). These observations were valid for various concentrations of rGO, which ranged from 0.1 to 100 mg/L. 

In order to gain insights into different behavior of bacteria in these experiments, we investigated the interaction of *E. coli* with rGO flakes in both NB and PS media. We observed instant flocculation when bacteria were introduced into high-concentration suspensions of rGO in both PS and distilled water. However, no such effect was observed when bacteria were added to suspensions of rGO in NB. Confocal microscopy and scanning electron microscopy (SEM) confirmed the accumulation of bacteria on rGO flakes. Interestingly, the *E. coli*-rGO flocculates were observed regardless of whether the bacteria were alive or dead. This observation, combined with the zeta potential measurements of the rGO suspensions before and after mixing with bacteria, which showed a significant drop from 40 to 14–15 mV, indicates the electrostatic nature of the interaction of graphene and bacteria, which could be mediated via hydrogen bonding between the cell walls of *E. coli* and rGO functionalities. 

The microscopic and zeta potential studies not only demonstrate the difference between the interaction of *E. coli* and rGO in NB and PS media but also suggests the possibility of using rGO for filtration and bacterial removal. Overall, the potential of graphene-based structures for water purification is widely recognized and a number of studies showed the possibility of using graphene and its derivatives in filter elements [19,20,21], which could employ the formation of conjugates of graphene-based materials and bacteria [22]. In our experiments, filtration of the suspensions of *E. coli*-rGO flocculates nearly completely removed live and dead bacteria from the filtered liquids, demonstrating the possibility of using rGO for the removal and separation of biological contaminants. 

## 2. Materials and Methods 

### 2.1. Synthesis of rGO 

All chemicals were purchased from Sigma-Aldrich (St. Louis, MO, USA) and used as received. GO was synthesized as described in our previous work [23], following the general procedure by Marcano et al. [24]. The reduction of GO to rGO was performed using ascorbic acid [25,26]. L-ascorbic acid (500 mg) was added to 250 mL of a GO aqueous dispersion (0.2 mg/mL) under vigorous stirring. The stirring was continued for three days at room temperature, and the reaction progress was monitored by UV-vis spectroscopy. After three days of stirring the color of the mixture changed from light amber to black. The reaction mixture was filtered with ethanol and water, and then dried under vacuum. Four rGO suspensions in deionized water at concentrations of 0.1, 1, 10 and 100 mg/L were prepared and used in the following fluorescence experiments. 

### 2.2. Materials Characterization 

Raman spectroscopy was performed using a DXR Raman microscope (Thermo Fisher Scientific, Waltham, MA, USA) with a 532 nm excitation laser and a 100× objective. SEM of rGO flakes on Si/SiO_2_ substrates was performed using a Supra 40 field-emission scanning electron microscope (Carl Zeiss AG, Oberkochen, Germany). SEM images of *E. coli*-rGO aggregates were recorded on a Vega 3 scanning electron microscope (Tescan, Brno, Czech Republic). Atomic force microscopy (AFM) was performed using a SmartSPM 1000 scanning probe microscope (AIST-NT, Novato, CA, USA). For AFM analysis, a droplet of an aqueous rGO suspension was deposited on a Si/SiO_2_ substrate and dried in air. X-ray photoelectron spectroscopy (XPS) was performed using a K-Alpha X-ray photoelectron spectrometer (Thermo Fisher Scientific, Waltham, MA, USA) with a monochromatic Al Kα (1486.6 eV) X-ray source.

### 2.3. E. coli Biofluorescence Tests

We used recombinant GFP expressing *E. coli,* which was transformed with pRSET-emGFP plasmid containing an ampicillin-resistant (amp^R^) gene using the standard electroporation procedure. The culture was used as a model system to investigate the antibacterial activity of rGO. Similar bacterial tests were widely used in the evaluation of toxicity of nanomaterials [27,28,29].

Bacteria were grown and tested in nutrient broth (NB) supplemented with ampicillin. 1 mL of overnight culture was grown in NB and then inoculated in 100 mL of fresh NB at 37 °C for 3 h. 50 mL of the culture was concentrated by centrifugation at 5000 *g* for 5 min. The culture was washed three times using a physiological saline solution (PS; 9 g/L NaCl aqueous solution) and centrifuged at 3000 *g* for 3 min. Optical density at 660 nm (OD660) of the resulting suspension of *E. coli* in PS was approximately 0.13, as measured using a Synergy H1 microplate reader (BioTek Instruments, Winooski, VT, USA). 

In a typical fluorescence experiment, 5 µL of the GFP *E. coli* suspension in PS was mixed with 200 µL of a culture medium (NB or PS) followed by the addition of 45 µL of an aqueous suspension of rGO with a concentration of 0.1, 1, 10 or 100 mg/L. After the addition of rGO to GFP *E. coli* suspensions, the behavior of bacteria was monitored by their green fluorescence at 528 nm using a microplate reader with an excitation at 485 nm. The fluorescence of GFP *E. coli* in different media was monitored at 37 °C for 17 h. The presence of rGO, which absorbs light in the entire visible range of spectrum, in the *E. coli* suspensions was taken into account for the correction of the fluorescence values. Each fluorescence kinetics experiment was performed at least 10 times, and the averaged results are presented. The analysis of statistically significant differences within the treated groups was carried out using the analysis of variance (ANOVA) for a single factor with further application of the Tukey’s multiple analysis of variance with a family error rate of 0.05.

### 2.4. Investigation of Interactions of E. coli with rGO

Optical microscopy was carried out on a Biolam M-1 microscope (LOMO, St. Petersburg, Russia) using the Ziehl–Neelsen stain. Confocal fluorescent microscopy measurements were performed on a TCS SP8 X CLSM setup (Leica Microsystems, Wetzlar, Germany) using the default settings for GFP (excitation at 488 nm, emission detection in the 500–600 nm range). Zeta potentials of dispersions were measured using a Zetasizer Nano ZS instrument (Malvern Instruments, Malvern, United Kingdom).

The suspensions for optical photographs were prepared by mixing 0.18 mL of 100 mg/L rGO solution with 0.02 mL of another solution (PS, *E. coli* in PS, dead *E. coli* in PS, NB, *E. coli* in NB, dead *E. coli* in NB). For the preparation of samples of dead *E. coli* in NB and PS, the bacterial suspensions were placed in a boiling water bath for 10 min.

## 3. Results

### 3.1. Characterization of rGO 

Considering the dependence of the toxicity of graphene-based materials on their characteristics, such as chemical composition, flake size and surface functionalization [15,16,17,18], we first performed a detailed materials characterization of rGO, which was used in this study. In general, GO could be reduced to rGO using a variety of chemicals such as hydrazine, hydroxylamine and sodium borohydride [30]. However, GO and related materials are known for their sorption ability [31], and when a toxic chemical, such as hydrazine [24,32], is used to produce rGO, there is a possibility that traces of a reducing agent or its derivative remain in a rGO sample and later affect the results of a nanotoxicity experiment. Therefore, for reducing GO to rGO in this study we specifically chose ascorbic acid [25,26], which is nontoxic and its possible traces were not expected to cause any inhibitory effects in experiments with *E. coli*.

**Figure 1:**
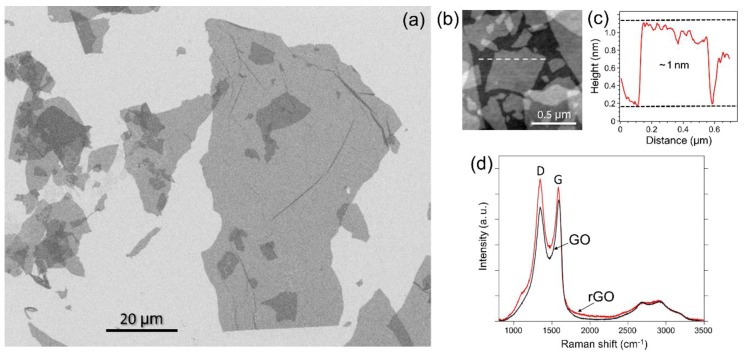
Characterization of graphene oxide (GO) and reduced graphene oxide (rGO). (**a**) Scanning electron microscopy (SEM) image of rGO flakes on a Si/SiO_2_ substrate. (**b**) Atomic force microscopy (AFM) image of rGO flakes on a Si/SiO_2_ substrate. (**c**) AFM height profile across the rGO flake, which was measured along the dashed line in panel (b). The flake has a height of about 1 nm. (**d**) Raman spectra of GO and rGO.

Characterization of rGO deposited on a Si/SiO_2_ substrate by scanning electron microscopy (SEM) and atomic force microscopy (AFM) revealed that the flakes had a wide size distribution with their lateral dimensions ranging from about 50 nm to several µm. SEM image in Figure 1a shows several rGO flakes on Si/SiO_2_ with the largest flake in the field of view being ~ 100 µm long. Smaller rGO flakes are shown in the AFM image of another area of the sample in Figure 1b. Figure 1c shows a representative height profile measured across one of the rGO flakes in Figure 1b. The flake has a height of about 1 nm, which is consistent with previous reports for rGO monolayers [33].

The Raman spectra of GO and rGO (Figure 1d) are also consistent with prior literature reports [34,35,36,37]. These spectra exhibit two broad peaks at about 1586 and 1345 cm^−1^, which are known as D and G bands, respectively [38]. It was previously demonstrated that in GO the intensity of the G band is slightly higher than the intensity of the D band, but upon the GO reduction the D band becomes more intense than the G band [34,35,36,37]. This previously established trend can also be seen in Figure 1d in the Raman spectra of GO and rGO prepared in this work.

**Figure 2:**
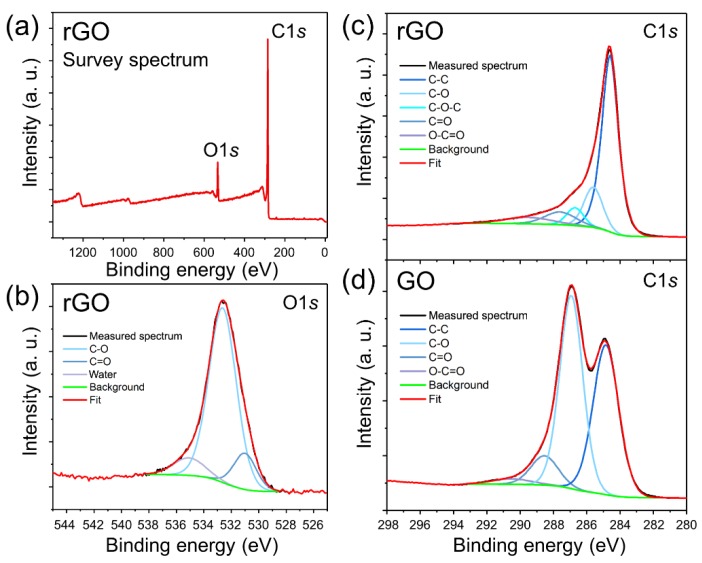
X-ray photoelectron spectroscopy (XPS) characterization of GO and rGO. (**a**) XPS survey spectrum of rGO. **(b)** XPS O*1s* spectrum of rGO. **(c)** XPS C*1s* spectrum of rGO. **(d)** XPS C*1s* spectrum of GO.

The reduction of GO to rGO was confirmed by X-ray photoelectron spectroscopy (XPS). The survey spectrum of rGO (Figure 2a) shows the presence of carbon (C*1s*) and oxygen (O*1s*) and no traces of surface contamination. The XPS O*1s* and C*1s* spectra of rGO and their deconvolutions are shown in Figure 2b,c, respectively. A comparison of XPS C*1s* spectra of rGO (Figure 2c) and GO (Figure 2d) shows the removal oxygen-containing functionalities in GO upon reduction using ascorbic acid. The XPS C*1s* core level spectrum of rGO predominantly demonstrates *sp*^2^ hybridized carbon atoms (284.6 eV) with considerably smaller contributions from carbon bonded to different oxygen-containing functional groups, such as hydroxyls (285.6 eV), epoxides (286.7 eV), carbonyls (287.6) and carboxyls (289.5 eV). Deconvolution of the XPS C*1s* core level spectrum of GO produces four peaks positioned at 284.85 eV, 286.93 eV, 288.53 eV and 290.52 eV, which were assigned to *sp*^2^ carbon, C–O, C=O, and O–C=O, respectively (Figure 2d). The carbon components were assigned according to the reported XPS spectra of GO and rGO samples containing the same oxygen-containing functional groups [34,36,39,40]. The quantitative XPS analysis of the samples shows that the C/O atomic ratio increases from 1.83 in GO to 9.37 in rGO, respectively, which is consistent with the XPS results reported for the rGO produced by GO reduction with ascorbic acid [26]. The fact that some oxygen-containing functionalities still remain in rGO is consistent with its solubility in water and will be important for explaining the results of nanotoxicity and aggregation experiments.

Overall, the results of materials characterization provided in Figure 1 and Figure 2 are consistent with other literature reports on rGO. The rGO material consists of well-exfoliated uniform monolayer flakes (Figure 1a–c) with minor amounts of oxygen-containing functionalities (Figure 2).

### 3.2. Bacterial Fluorescence Studies

**Figure 3:**
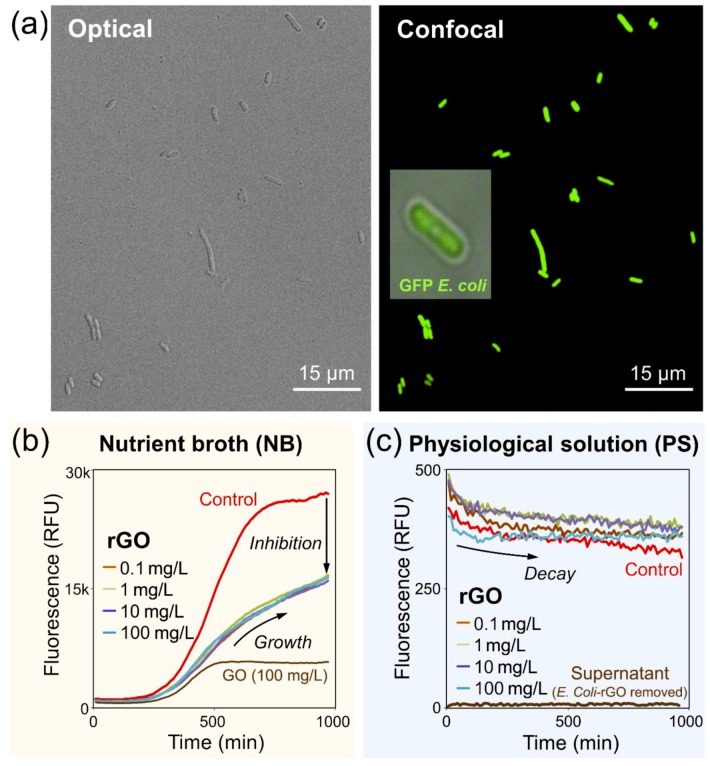
Effect of rGO on *E. coli* bacteria in nutrition broth (NB) and physiological solution (PS) media. (**a**) Optical (left) and confocal microscopy (right) images of suspensions of green fluorescent protein (GFP) expressing *E. coli* bacteria. The inset in the right panel shows magnified image of an individual *E. coli* by confocal microscopy. (**b**,**c**) Fluorescence of GFP *E. coli* bacteria in NB (**b**) and PS (**c**) after the addition of rGO at concentrations ranging from 0.1 to 100 mg/L. The red curves in (**b**,**c**) show the fluorescence of *E. coli* in control samples, in which the bacteria were measured in NB and PS, respectively, without the addition of rGO. The brown curve in (**b**) shows fluorescence of GFP *E. coli* bacteria in NB after the addition of 100 mg/L GO solution. The brown curve in (**c**) shows no fluorescence from the supernatant decanted from the *E. coli*-rGO colloidal solution in PS*,* suggesting that the bacteria aggregated with the rGO sheets and were removed from solution. The fluorescence in panels (**b**) and (**c**) is shown in the same relative fluorescence units (RFU).

For this study, we used green-fluorescent *E. coli* bacteria, which are shown in optical and confocal microscopy images in Figure 3a. The images were collected simultaneously, and their comparison shows that all bacteria exhibited green fluorescence. The rGO dispersions at different concentrations were added to *E. coli* dispersed in NB and PS culture media, and then the behavior of bacteria was monitored by fluorescence spectroscopy.

Figure 3b shows that in the NB growth medium the fluorescence of *E. coli* significantly decreased (almost 2-fold) for all concentrations of rGO compared to the control sample (red curve) to which the rGO was not added. However, just as in the control experiment, in the presence of rGO the bacteria continued to grow, albeit at lower rates.

Compared to the experiments performed in NB, the dispersions of *E. coli* in PS generally showed much lower fluorescence intensities (Figure 3c), which is associated with the absence of nutrients in PS compared to NB. Also, unlike the case of NB, where the introduction of rGO had a strong inhibitory effect on the bacterial growth, in PS, the administration rGO had little effect on the behavior of GFP *E. coli* (Figure 3c), and then the fluorescence of GFP *E. coli* decreased with time due to the absence of nutrients.

**Figure 4:**
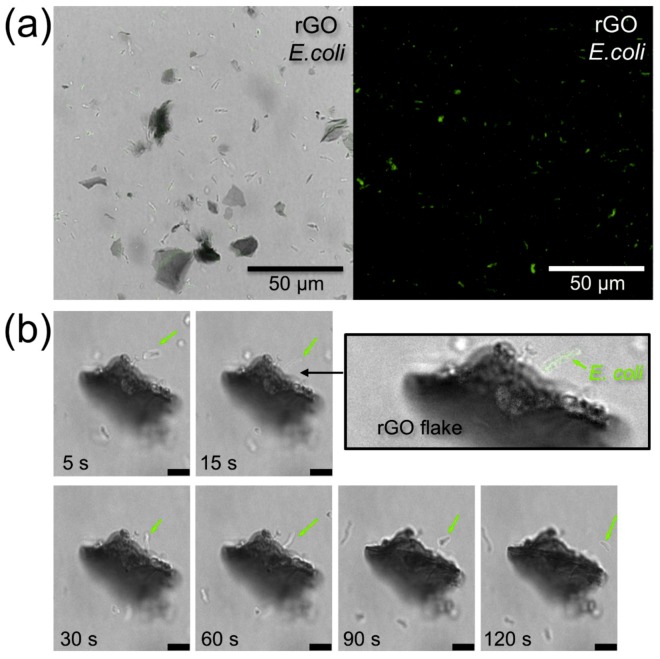
Dynamics of the interaction of rGO and bacteria dispersed in PS. (**a**) Optical (left) and confocal (right) images of rGO flakes (10 mg/L) and *E. coli* bacteria dispersed in a droplet of PS. Live bacteria emit a green fluorescent signal. (**b**) A series of confocal microscopy images illustrating the interaction of an individual *E. coli* shown by the green arrows with a rGO flake. Scale bars are 5 μm.

The fact that bacteria remain alive after the addition of rGO can also be verified by optical and confocal microscopy. Figure 4a shows optical (left panel) and confocal (right panel) microscopy images of a droplet of a suspension of rGO flakes (10 mg/L) and *E. coli* bacteria in PS 15 min after mixing. Large flakes of rGO dispersed in PS are visible in the optical image, while the green fluorescence of GFP *E. coli* in the confocal microscopy image shows uniform distribution of bacteria in the droplet. The emission of GFP *E. coli* confirms that the bacteria are alive in the presence of rGO flakes.

Figure 4b shows a series of confocal microscopy images illustrating the interaction of an individual *E. coli* with a rGO flake. The bacterium approaches the flake and then leaves continuing its green fluorescence, indicating that it remains alive and was not damaged by the contact with the flake. This observation indicates that a contact with graphene does not necessarily damage the cell membrane and result in bacterial death, as discussed in previous studies [7,41,42,43]. However, other toxicity mechanisms could manifest in a particular experiment, which could involve adsorbed rGO contaminants [14,44] and a variety of other factors [4,5].

Overall, in both media—NB and PS—the bacteria exhibited qualitatively same behavior with and without rGO, suggesting that the presence of nutrients was a more important factor for bacteria growth than the addition of rGO. Yet, rGO had very different effects on *E. coli* in NB and PS, strongly decreasing the fluorescence of bacteria in NB and showing little effect on it in PS compared to control experiments (Figure 3b,c).

While the observed effects of rGO on *E. coli* in different media are likely the result of a complex interplay of multiple physicochemical phenomena, they could in part be rationalized through the interaction of nutrients (if present) and bacteria with the dispersed rGO sheets. Graphitic structures are known for their sorption ability, and accumulation of the nutrients on dispersed rGO sheets could be the reason for the reduced growth rate of *E. coli* in NB if rGO is introduced (Figure 3b). In this case, small biomolecules present in NB likely saturate the surface of rGO sheets, minimizing the interaction of bacteria with rGO. Conversely, in the PS medium, where no such nutrients are present, the conditions are more favorable for the interaction between bacteria and rGO. The rGO sheets contain a variety of oxygen-containing functionalities, such as hydroxyl, epoxy and carboxyl groups [24,34], which engage in inter- and intra-molecular hydrogen bonding [45]. The cell walls of *E. coli* consist of a variety of biomolecules [46,47] containing similar oxygen functionalities, which form hydrogen bonds as well [48]. Because of the presence of these oxygen functional groups in both rGO and cell membranes, the aggregation of rGO sheets and bacteria mediated via hydrogen bonding is expected. A number of studies have further shown that bacteria can consume oxygen from GO sheets [49,50], which could be the reason for the possible slight activation of *E. coli* in PS in the presence of rGO (Figure 3c).

Previous studies considered numerous factors affecting the toxicity of graphene-based materials toward bacteria, such as flake size [41], surface functionalization [51], number of layers [52], coagulation and dispersity [22]. Our results demonstrate that the nature of the culture medium also plays an important role in nanotoxicity experiments and can substantially affect the conclusions regarding the antibacterial properties of rGO. This conclusion is consistent with the previously reported data for GO [43] and is likely valid for a variety of other graphene-based materials.

### 3.3. rGO-Bacteria Interactions

**Figure 5:**
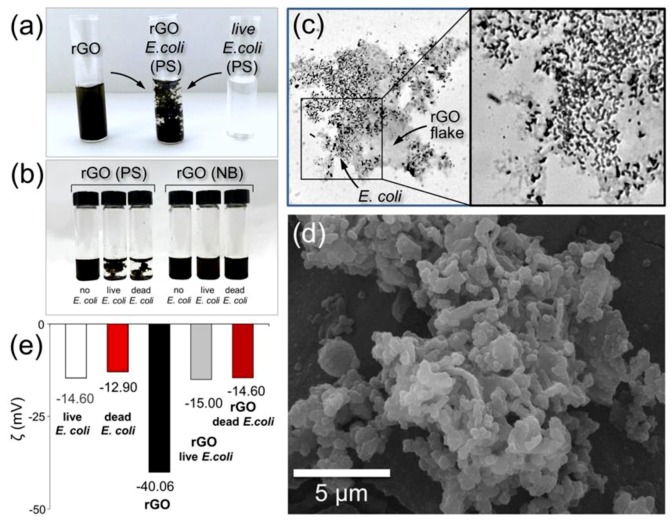
Interactions between rGO and bacteria dispersed in PS. (**a**) Optical photograph of a 1 g/L rGO aqueous suspension (left), a bacterial suspension in PS (right) and a flocculate structure produced by mixing the rGO and *E. coli* suspensions (middle). (**b**) Optical photograph of the suspensions produced by mixing rGO solution with (from left to right) PS, rGO solution with alive bacteria in PS, rGO solution with dead bacteria in PS, rGO solution with NB, rGO solution with alive bacteria in NB, rGO solution with dead bacteria in NB. (**c**) Optical photographs of *E. coli* bacteria localized on a rGO flake. (**d**) SEM image of *E. coli*-rGO aggregates. (**e**) Zeta potentials of colloidal suspensions of live and dead *E. coli* bacteria in PS, rGO and mixtures of rGO with live and dead *E. coli*.

In order to substantiate the hypothesis that the difference in the effects of rGO on *E. coli* in NB and PS media is related to adsorption of bacteria on rGO sheets, we performed microscopic and zeta potential studies of rGO-bacteria interactions. These interactions could be easily visualized by mixing a highly concentrated rGO solution (1 g/L) with a bacterial suspension in PS, as shown in Figure 5a. The mixing of two suspensions results in an immediate formation of a stable flocculate structure (Figure 5a,b), which could be dispersed by a sonication but then quickly reforms. Based on the visual observations, similar 1g/L solutions of GO exhibited an even stronger flocculation with *E. coli* in PS producing larger and visibly denser aggregates.

A droplet of an *E. coli*-rGO colloidal solution in PS was deposited on a glass microslide and dried in air for a microscopy analysis. Optical photographs in Figure 5c show that the bacteria are localized on a rGO flake but not on a bare substrate, suggesting strong interaction between bacteria and rGO sheets, which is likely mediated by hydrogen bonding. The bacteria/rGO aggregates were also visualized by SEM (Figure 5d).

Interestingly, no flocculation was observed when rGO suspensions were mixed with suspensions of *E. coli* in NB (Figure 5b), and in the optical and SEM images the rGO flakes and bacteria were observed separately. In principle, the presence of salt in PS could affect the aggregation of graphene-based materials, as was shown in previous studies [53]. However, in the control experiment, we did not observe any flocculation when rGO was mixed with pure PS without *E. coli* (Figure 5b) suggesting that the presence of bacteria is important for the aggregation of rGO (Figure 5a), and this is not only the effect of the dissolved salt. The conclusion that the salt is not a determining factor for the aggregation of rGO and *E. coli* is also supported by the fact the aggregates formed in suspensions of rGO and bacteria in distilled water.

Overall, these observations are consistent with the results of fluorescent studies shown in Figure 3b,c. The flocculation is likely caused by the hydrogen bonding between the oxygen-containing groups in rGO sheets and biomolecules comprising the cell walls of *E. coli* bacteria. The flocculation is observed for the bacteria dispersed in PS, while in NB the interaction between the rGO and *E. coli* is inhibited as the surface of rGO sheets is saturated by the nutrient biomolecules. These observations suggest that the inhibition of the GFP *E. coli* fluorescence in NB should be stronger for GO than for rGO considering that the former contains more oxygen-containing functional groups that could interact with nutrients. This is illustrated by the brown curve in Figure 3b for the 100 mg/L GO solution, which shows a considerably lower fluorescence of GFP *E. coli* than the 100 mg/L rGO solution.

Unlike other studies, we also performed experiments with dead bacteria. Similar flocculate structures were formed when rGO suspensions were mixed with the suspensions of dead bacteria in PS (Figure 5b), which was noted for the first time and does not confirm the opinion about bacterial biofilms as the basis for the formation of graphene-bacteria conjugates [11,22]. Measurement of the zeta potential of the particles in the rGO suspensions before and after mixing with bacterial cells (alive or dead) showed its decrease from 40 to 14–15 mV (Figure 5e), which indicates the electrostatic nature of the formation of *E. coli*-rGO flocculates.

When we decanted a supernatant from the freshly prepared *E. coli*-rGO colloidal solution in PS, thus removing the dark *E. coli*-rGO flocculates, the visibly clear solution did not exhibit any detectable fluorescence signal (see the brown curve in Figure 3c), suggesting that the bacteria were aggregated with the rGO sheets. Likewise, filtration of the suspensions leads to almost complete removal of bacteria from the filtered liquids, which indicates the total character of the conjugation process and highlights the potential of rGO for filtration and separation of biological contaminants, regardless of whether they include live or dead microorganisms.

## 4. Discussion

While previous studies showed that the toxic effects associated with rGO depend on a large number of factors, which include the flake size, surface functionalization and concentration among other parameters, here we demonstrate that the type of culture medium is also an important factor that could affect the outcome of a nanotoxicity experiment. We performed experiments involving the same dispersions of rGO and same fluorescent *E. coli* bacteria and found that rGO inhibited the growth of bacteria in a nutrition medium but had little effect on the behavior of *E. coli* in a physiological saline solution. These observations were valid for various concentrations of rGO, which ranged from 0.1 to 100 mg/L.

While the observed effects of rGO on *E. coli* in different media are likely the result of a complex interplay of multiple physicochemical phenomena, they could in part be rationalized through the adsorption of nutrients (if present) and bacteria on the dispersed rGO sheets. The interaction of bacteria and rGO is likely mediated via hydrogen bonding between the biomolecules forming the cell walls of *E. coli* and oxygen-containing groups in rGO sheets. We investigated the interaction of *E. coli* and rGO and found it to also be medium dependent. In physiological saline solution, as well as in distilled water, *E. coli* and rGO instantly aggregate, and microscopy reveals accumulation of bacteria on rGO flakes. Furthermore, this aggregation of rGO and *E. coli* was observed regardless of whether the bacteria were alive or dead. No aggregation was observed in nutrition media. Filtration of the aggregate suspensions led to nearly complete removal of bacteria from filtered liquids, which highlights the potential of rGO for filtration and separation of biological contaminants, regardless of whether they include live or dead microorganisms.

Interestingly, the effect of culture medium on the toxicity of GO toward *E. coli* was also considered in the study by Hui et al., who also attributed the observed phenomena to the molecular adsorption on GO sheets [43]. Although this study focused on GO instead of rGO and operated in a higher concentration range (80–300 mg/mL, which could be related to the lower optical absorption of GO compared to rGO), the observations made for GO [43] and rGO (Figure 3b,c) overall agree with each other. A small decrease in the amount of *E. coli* in saline solutions was observed several hours after the addition of 80 μg/mL GO [43] and for all rGO concentrations tested in this work (Figure 3c). Interestingly, the work by Hui et al. shows that at higher concentrations, such as 200 μg/mL, GO becomes highly toxic to *E. coli* in saline solutions [43]. On the other hand, the present work demonstrates that at lower rGO concentrations down to 0.1 μg/L all tested solutions showed similar fluorescence decays to the control experiment, suggesting that they are likely caused not by the toxic effect of rGO but rather the overall lack of nutrients. Also, in cases of both GO [43] and rGO (Figure 3b), the bacteria grow with the addition of nutrients. While a direct comparison between the GO and rGO experiments should be done cautiously, it appears that the two studies are not contradictory and complement each other by covering different materials and concentration ranges.

In summary, the type of culture medium is shown to strongly affect the antibacterial properties and bacterial filtration ability of rGO in experiments with *E. coli* bacteria and is likely an important factor for consideration in nanotoxicity studies of other graphene-based materials.
